# Ultrastructure of the Endoplasmic Reticulum in Eukaryotic Microalgae

**DOI:** 10.1111/jeu.70030

**Published:** 2025-07-30

**Authors:** Ursula Goodenough, Robyn Roth

**Affiliations:** ^1^ Department of Biology Washington University St. Louis Missouri USA; ^2^ Center for Cellular Imaging Washington University School of Medicine St. Louis Missouri USA

**Keywords:** chloroplast, endosymbiosis, Golgi, lipid body, nuclear envelope, quick‐freeze deep‐etch electron microscopy

## Abstract

The endoplasmic reticulum (ER) is a large and highly dynamic component of the eukaryotic endomembrane system. In eukaryotic microalgae, it plays six distinct roles: (1) It envelopes the chromatin to form the *nucleus*. (2) It forms cisternae in the cytoplasm, some of which scaffold the synthesis of proteins destined for incorporation into membranes or for secretion. (3) It associates with *Golgi* cisternae to scaffold the synthesis of glycosylated proteins. (4) It associates with the *plasma membrane* to mediate the synthesis and secretion of hydrophobic molecules. (5) It mediates the synthesis of cytoplasmic *lipid bodies*. (6) In lineages harboring complex plastids of red algal ancestry, it forms the *chloroplast ER*, which envelops the primary chloroplast envelope. In this review, these systems are illustrated using the quick‐freeze deep‐etch electron microscopy (QFDEEM) technique, which lifts up the topological configurations adopted by this gossamer system. A key finding is that in all the complex microalgae examined except dinoflagellates, the inner nuclear envelope membrane associates directly with the plastid‐contiguous membrane of the chloroplast ER at foci designated as chloroplast‐nuclear junctions. These junctions may play a role in regulating the maintenance and physiology of the complex organelles.

## 1 Introduction

The endoplasmic reticulum (ER) is created by a membrane that folds back on itself to form a single enclosure with a tiny lumen (30–50 nm in diameter), a separation mediated by several proteins that span the lumen (Shen et al. 2019). In microalgae, the system usually adopts a cisternal topology https://www.britannica.com/science/endoplasmic‐reticulum although tubules are occasionally encountered; shape has been shown to be mediated by several protein families (Hu et al. 2011; Kontou et al. 2022; Perkins and Allan 2021; Sandoz et al. 2023; Schwarz and Blower 2016; Shemesh et al. 2014; Westrate et al. 2015). Each cistern has two “sides” that face one another. In some cases, the apposing membranes are symmetric, performing the same function, as in textbook diagrams of ribosome‐studded “rough” ER. In other cases, they are asymmetric, each side performing distinctive functions in localized or extended domains. A cistern can also form an asymmetric envelope that surrounds a second cellular domain such as chromatin.

The ER is present in all modern eukaryotes and shares a set of protein components (Kontou et al. 2022); hence, it was presumably also present in the Last Eukaryotic Common Ancestor (LECA). It was first visualized and named in mammalian preparations (Porter 1953; Palade 1956), and has been extensively analyzed in multicellular lineages where it participates in numerous somatic cell specializations such as calcium storage/release systems in muscle cells, plasmodesmata in leaf cells, and smooth ER in steroid‐secreting cells (Obara et al. 2023), indicating its capacity to be programmed for numerous functions.

In this review, we analyze ER ultrastructure in a wide range of unicellular eukaryotic algae, including red and green primary algae and algae harboring complex plastids with a red algal origin such as diatoms, crysophytes, cryptophytes, and dinoflagellates, using images acquired during our study of their lipid biodiesel precursors. We also present some examples from fungi for comparison. The images were obtained using the quick‐freeze deep‐etch (QFDE) EM technique, which provides panoramic views of ER membranes that are not attainable with traditional EM techniques, thereby deepening our understanding of cisternal dynamics.

## 2 Methods

Most of the algal specimens were cultured in the labs listed in Acknowledgments and overnight‐shipped to our lab, usually in log phase. Upon arrival, samples were examined by light microscopy to ascertain that the cells appeared healthy. *Chlamydomonas reinhardtii* , *Botryococcus braunii* , *Cladonia grayi* , *Cyanidioschyzon merolae, Nannochloropsis gaditana*, *Ochromonas danica* , and *Polytomella parva* were cultured in our lab.

For quick‐freeze deep‐etch (QFDE) electron microscopy, pelleted live cells were placed on a cushioning material and frozen by forceful impact against a liquid‐He‐cooled copper block mounted within a Heuser Cryo‐Press Quick‐Freezing Device (ValiantInstruments, Ellisville, MO 63011, USA). The frozen material was transferred to liquid nitrogen, fractured under vacuum, etched at −100°C for 2 min, and Pt/C rotary‐replicated under vacuum using a Balzers BAF 400 D Freeze Etching System (Balzers AG, Lichtenstein), as described in Heuser (1981) and Roth and Goodenough (2021). The platinum layer, 2–3 nm thick, was stabilized by the overlying carbon layer. Pt/C replicas were digested with chromosulfuric acid for 15 min to remove cellular material and examined with a JEOL electron microscope, model JEM 1400 (JEOL, Peabody, MA 01960), equipped with an AMT (Advanced Microscopy Techniques, Woburn, MA 01801, USA) V601 digital camera. The figures show photographic reversals: platinum deposits, which block the electron beam and are dark in the original images, appear white.

Features of the QFDE technique that are relevant to an understanding of the figures include the following. When the fracture plane passes within the oily interior of a frozen membrane, the membrane is split into two leaflets: a P leaflet adjacent to the cytoplasm and an E leaflet adjacent either to the cell exterior (plasma membrane) or to the interior of an organelle. In cases where the fracture removes the P leaflet, an E face is exposed; when the fracture removes the E leaflet, a P face is exposed. During fracture, membrane‐spanning proteins tend to remain associated with the P leaflet, generating intramembranous particles (IMPs), while the E leaflet typically carries the holes/pits that remain after the IMPs are pulled out. Etching sublimes a thin layer of water off the fractured surface, exposing more detail but in some cases destabilizing structural integrity.

## 3 Overview

We begin with the nuclear envelope that encloses chromatin and is traversed by nuclear pore complexes (Chou et al. 2021). The outer nuclear envelope membrane (oNM) is generally studded with ribosomes, mediated by the ribosome binding protein p180 (Benyamini et al. 2009), while the ribosome‐free inner nuclear envelope membrane (iNM) is in contact with chromatin‐related proteins and lamin‐related filaments (Odell and Lammerding 2023).

The algal oNM bulges out at discrete sites to become coextensive with ER cisternae in the cytoplasm, also called peripheral ER. These membranes initially have ribosomes on both sides, and while some continue to maintain this topology, they do not form the stacks of rough ER encountered in textbook diagrams. Instead, most differentiate in one of the following ways:

- *Golgi ER*: Cisternae extend into the cell interior, where one side loses ribosomes and feeds coated vesicles into localized Golgi domains while the other side retains ribosomes.
- *Lipid Body ER*: Cisternae participate in the formation of lipid bodies throughout the cytoplasm, where both sides display this capability.
- *Cortical ER*: Cisternae extend to the cell cortex, where the outward‐facing side loses ribosomes and makes contact with the plasma membrane while the inward‐facing side retains ribosomes. In some cases, these cisternae adopt a reticulated topology and may be perforated by fenestrae.
- *Chloroplast ER*: The red and green algae harbor a “primary” chloroplast, derived from an endosymbiotic cyanobacterium, that is surrounded by a chloroplast envelope. The “complex” algal species considered here harbor a chloroplast derived from an endosymbiotic red alga, which retains its original envelope and acquires a second enveloping cisternum derived from the host ER. The plastid‐facing membrane of the chloroplast ER loses ribosomes and associates with the outer membrane of the chloroplast envelope, while the cytoplasm‐facing membrane retains ribosomes.

In the sections below, we consider these differentiations. Punctate contacts between the ER and other organelles, such as mitochondria and vacuoles, are also evident in our images and have been studied in yeast and animal cells (Almeida and Amaral 2020; Obara et al. 2024; Perkins and Allan 2021), where they have been implicated in the transfer of lipids (Wong et al. 2019; Wu, Carvalho, and Voeltz 2018). These are not, however, considered in this review.

## 4 Nuclear Envelope

**FIGURE 1:**
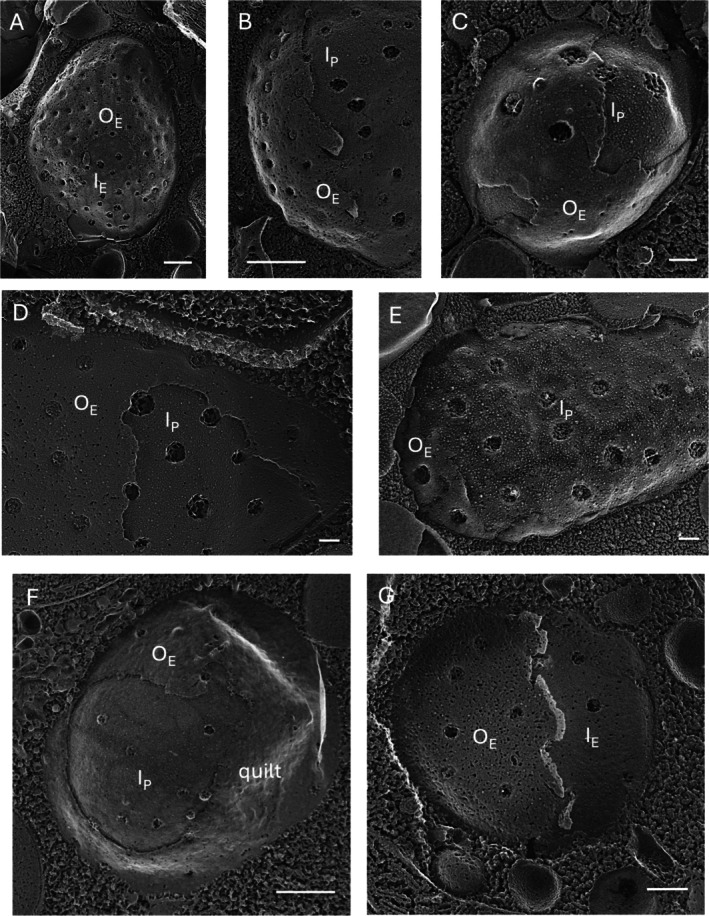
Nuclear envelopes and their pores in red and green algae. Pore morphology is vulnerable to fracturing/etching. (A) *Chlamydomonas reinhardtii*. (B) *Dunaliella salina*. (C) *Cyanidioschyzon merolae*. (D) *Polytomella parva*. (E) *Borodinellopsis texensis*. (F) *Auxenochlorella protothecoides.* (G) *C. reinhardtii*. I_E_, inner membrane E face; I_P_, inner membrane P face; O_E_, outer membrane E face; O_P_, outer membrane P face. Bars (nm): A, 500; B, 500; C, 100; D, 100; E, 100; F, 500; G, 250.

Figure 1 illustrates the oNM and iNM of the nuclear envelope in various green and red microalgae; Figure S1 shows several examples from complex algae and yeasts. The variability in nuclear pore density is evident. A fracture traveling through the oNM often exposes its E leaflet (O_E_) and then jumps down to the iNM to expose its P leaflet (I_P_). The P faces of both membranes carry the expected population of IMPs, while the E faces are more variable. In some cases, they carry the expected array of complementary pits (Figure 1B,D), but in many cases, they are destabilized by the etching process: the pits lose their integrity, and the face looks “soupy,” often adopting a wavy topology that we designate as quilted (Figure 1F,G, Figure S1 B,E,F). E faces that are unstable to etching have been documented in other algal organelles as well, most dramatically in the acidocalcisome (Goodenough et al. 2019).

## 5 Nuclear Bridges

The nuclear envelope has been shown in animals to be an extension of the ER that re‐assembles around chromosomes at the conclusion of each open mitosis (Deolal et al. 2024; Lu, Ladinsky, and Kirchhausen 2011). Since most of the algae under study engage in a closed mitosis, we have not observed this process.

**FIGURE 2:**
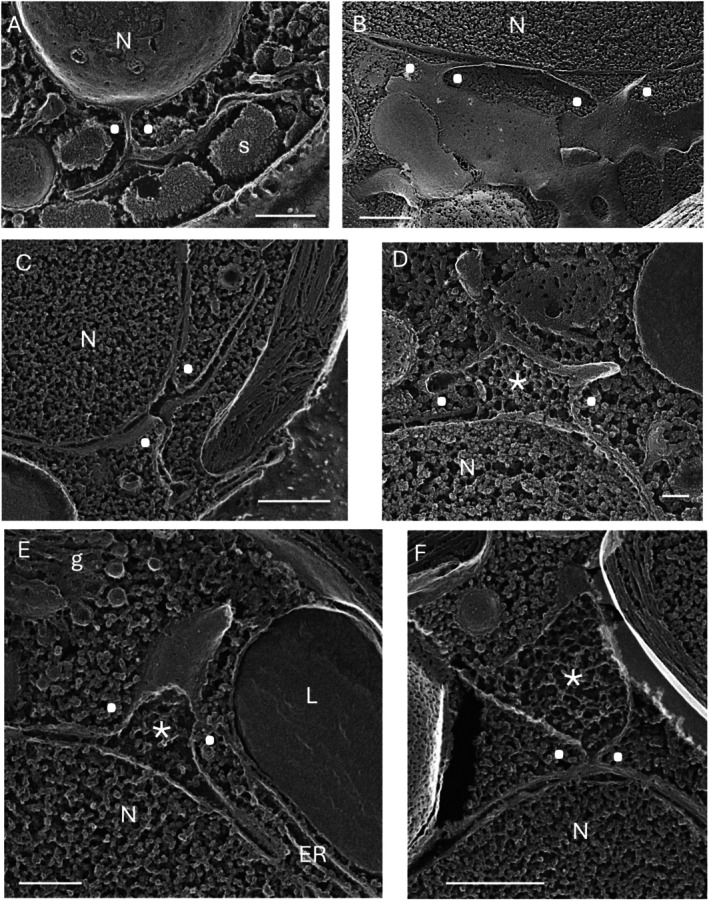
Bridges between outer nuclear envelope and cytoplasmic ER. (A) *Cyanidioschyzon merlolae*. (B) *Dunaliella salina*. (C–F) *Chlamydomonas reinhardtii*. ER, endoplasmic reticulum associated with a lipid body (L); asterisks, fibrillar material within bridge lumen; g, Golgi; N, nucleus; paired white dots, bridge; s, starch. Bars (nm) A, 250; B, 250; C, 500; D, 100; E, 250; F, 500.

Domains that bridge the envelope and cytoplasmic ER (Figure S2), herein called nuclear bridges but also called junctions (Deolal et al. 2024), involve the formation of a T‐shaped intersection (Figures 2 and S3B, paired dots, and Bragulat‐Teixidor et al. 2025). The ER in the bridge is in direct continuity with the ribosome‐studded oNE (Figure S3B); selective ribosome loss then accompanies various differentiation pathways that occur in the cytoplasm, as illustrated in Figure S3B. In mammals, the nuclear envelope displays a distinctive protein and lipid profile from cytoplasmic ER, where the bridges appear to be involved in maintaining this differentiation (Deolal et al. 2024), and bridge ultrastructure undergoes significant transformations during the course of the cell cycle (Bragulat‐Teixidor et al. 2025).

Figure 2D–F show examples from *Chlamydomonas* where the bridge lumen contains a fibrillar material (asterisks), of unknown function, that is also present in the lumen of Golgi‐associated ER (see later section). Since most of the bridges lack this material, the images support the possibility that the fiber‐filled bridges in Figure 2 are continuous with ER domains that generate the coated vesicles feeding Golgi systems (see next section). A previously published thin‐section (Figure S3B) further supports this interpretation. More generally, each bridge may be marked for a particular cytoplasmic destination and function.

## 6 Generic Cytoplasmic ER

Figure S2 shows images of generic cytoplasmic ER from various algae, presumably equivalent to rough ER (with rare exceptions (Figure S2E) ribosomes are difficult to discern in QFDE replicas). The P and E fracture faces are similar to their nuclear counterparts, including E faces vulnerable to etching and occasional quilting. The IMPs in the P faces of cytoplasmic and nuclear membranes presumably represent unique populations of transmembrane proteins that enable differentiated functions in specific locations, but we found no examples of a patterning in their size or distribution that suggest functional correlates, in contrast to the patterning that can be imposed on the algal plasma membrane (e.g., Goodenough et al. 2018).

## 7 Golgi Configurations

Stacks of Golgi cisternae are found in two configurations in eukaryotic microalgae.

In the textbook configuration, the stacks associate with an ER cisternum in the cytoplasm. In local regions, typically 0.5–1.0 μm in diameter (Figure S3A), one membrane lacks ribosomes and displays small coated vesicles that feed into the cis face of an adjacent Golgi, while the apposing membrane retains its ribosomes (Figure S3A,B). The lumen of the ER may dilate in these regions, and in *Chlamydomonas* it is filled with an unidentified fibrillar material (Figure S3, asterisks), noted earlier as present in some bridges (Figure 2D–F, asterisks), that is absent in other organisms. A given ER cisternum can give rise to several contiguous Golgi bodies (Figure S3A,C), but we have found no images showing Golgi associating with both apposing sides of an ER cisternum.

**FIGURE 3:**
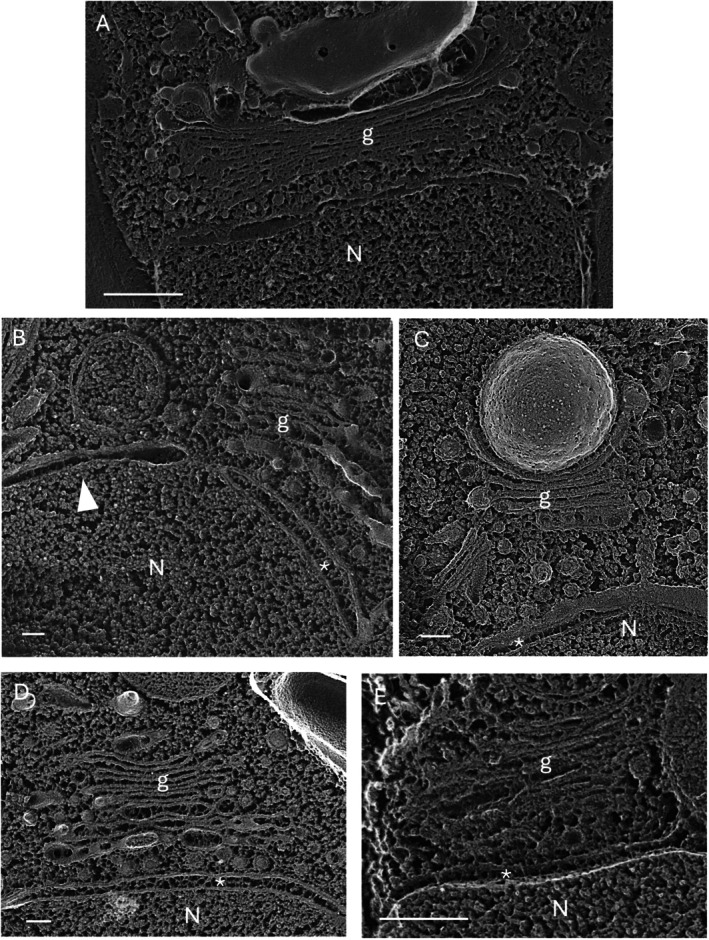
Golgi serviced by nuclear envelope. (A) *Ochromonas danica*. (B) *Chromera velia*. (C) *Auxenochlorella protothecoides*. (D) *C. velia.* E. *Phaeodactylum tricornutum*. arrowhead, contiguous envelope domain lacking filaments; asterisks, filaments inside the envelope domain servicing Golgi; G, Golgi; N, nucleus. Bars (nm): A, 500; B, 100; C, 100; D, 100; E, 250.

The second configuration, first documented in brown algae (Bouck 1965), arises when a localized domain of the oNM loses its ribosomes and forms coated vesicles that feed into Golgi stacks (Figure 3), creating what we call a nuclear Golgi. To our knowledge, this configuration has thus far only been observed in eukaryotic microalgae, including the apicomplexans that evolved from microalgae (Hager et al. 1999) and a few genera of the heterotrophic Stramenophiles (oomycetes) (Bracker et al. 1996). In all of these cases, the apposing membrane is the iNM, which interfaces with chromatin. Hence, both faces are ribosome‐free.

Filamentous material is present within the lumens of the nuclear envelope domains servicing Golgi domains (Figure 3, small asterisks), analogous to the fibrils within the Golgi‐serving cytoplasmic ER in *Chlamydomonas* (Figure S3, large asterisks) but distinctive in morphology. This material is absent from an envelope lumen adjacent to a nuclear Golgi lumen (Figure 3B, arrowhead), underscoring the ER's capacity for regional differentiation.

Curiously, a nuclear Golgi has been observed only once in our thousands of *Chlamydomonas* images; the Golgi are otherwise serviced by filament‐filled cytoplasmic ER cisternae that are bridged with the envelope (Figure S3B), where the trans face of the system faces the cell interior (Figure S3A). They have also not been observed in red algae, whose small Golgi are generally restricted to the cell periphery (Gant and Conti 1965).

That said, in many and perhaps most microalgae, the nuclear and ER membranes are both capable of transforming into discrete Golgi‐associated domains. Nothing is known about how these domains, in either location, come to be differentiated from the ribosome‐studded bilayer that surrounds them, nor whether they engage in processing distinctive populations of proteins.

## 8 Lipid Body Configurations

ER‐localized enzymes synthesize the vast majority of cellular lipids (reviewed in Jacquemyn, Cascalho, and Goodchild 2017). In addition, ER membranes participate in the formation of triacylglyceride‐containing lipid bodies (LBs) in the cytoplasm. A previous paper provides a QFDE analysis of this process in *Chlamydomonas* (Goodson et al. 2011), where the outer membrane of the chloroplast envelope also participates (seen as well in *Dunaliella* (Polle et al. 2020) but not in other microalgae).

Figure 2E and Figure S4 show LB formation in representative algae. The P face of the ER loses its resident IMPs when it interfaces with a LB: both fracture faces are totally smooth (Figure S4F and figure 9 of Goodson et al. 2011).

The independence of the two “sides” of an algal cisternum is illustrated in figure 8C of Goodson et al. (2011) where one membrane is servicing a LB and its apposing membrane is servicing a Golgi domain. Another QFDE study (Arakawa et al. 2022, figure 12C) shows a fungal cisternum where one membrane associates with a LB and the other with the plasma membrane. Figure 12A of Goodson et al. (2011) and Figure S4C,D show apposing ER membranes both servicing LBs, documenting that this capability can be symmetric. Figure S4E and 12B of Goodson et al. (2011) show that the oNM can also mediate LB formation, further illustrating the functional plasticity of this membrane.

Figure S4G shows a nascent lipid body being serviced by both cisternal and tubular ER elements; tubular ER participation in LB formation is also illustrated in figure 12A of Goodson et al. (2011). These are the only examples of a tubular ER configuration encountered in our large collection of microalgal QFDE images.

## 9 Cortical ER

ER with ribosomes on both membranes is often found in the cell periphery of microalgae (Johnson and Porter 1968; Kim and Archibald 2013), but a distinctive system, called cortical ER, retains ribosomes on one membrane while the other makes direct contact with the plasma membrane (Gallo et al. 2016; Hepler et al. 1990; Hirose et al. 2013; Ng et al. 2020).

**FIGURE 4:**
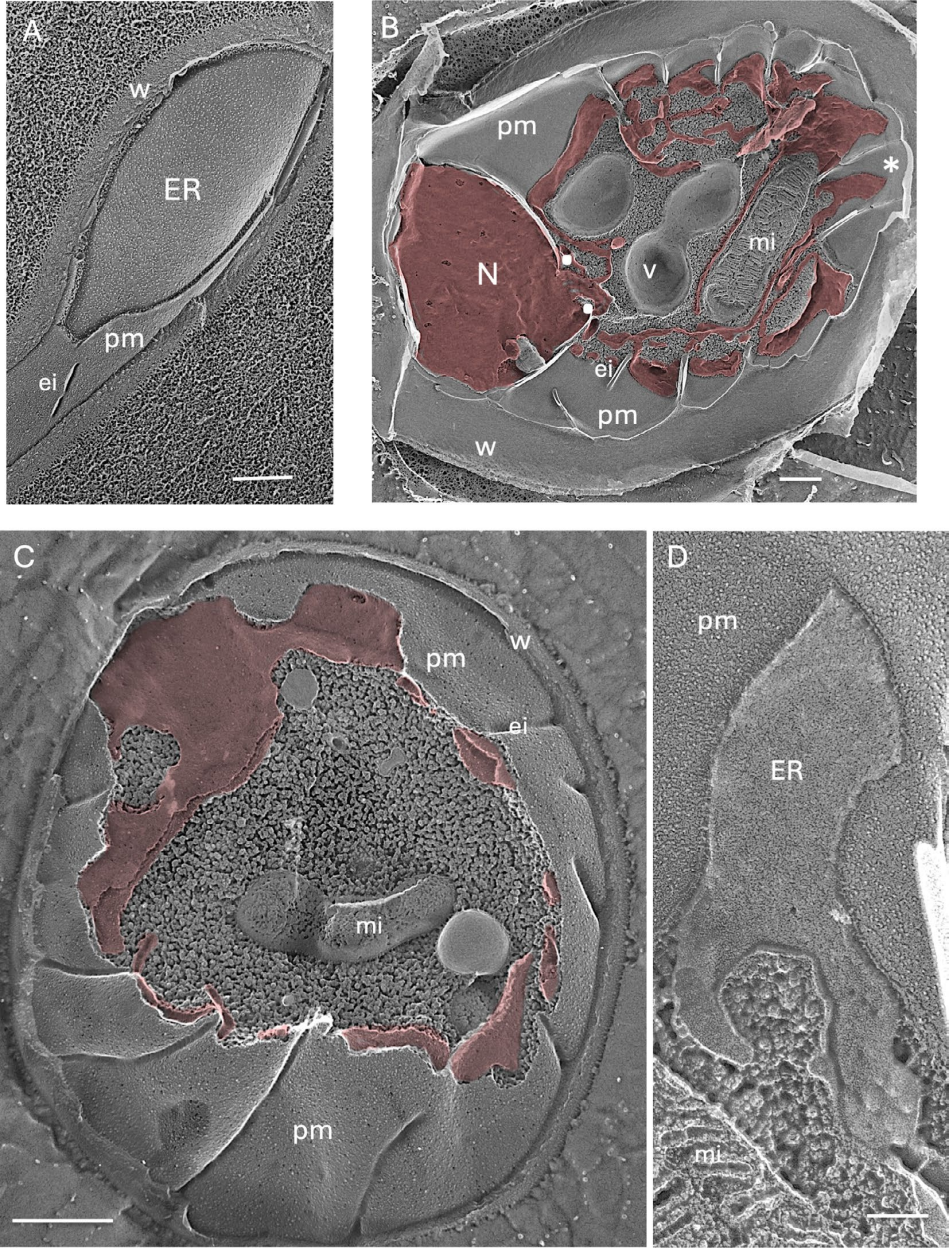
Cortical ER (A) Lichenogenic fungus *Myelochroa leucotyliza* incubated in culture media of algal symbiont (Arakawa et al. 2022). (B) *Polytomella parva* cyst, cortical ER in pink. (C) *Auxenochlorella protothecoides* exposed to decane, cortical ER in pink. (D) Enlargement of field marked with asterisk in Figure 4B. asterisk, field enlarged in Figure 4D; Ei, eisosome; mi, mitochondrion; N, nucleus; paired white dots, bridge; pm, plasma membrane; v, vacuole; w, wall. Bars (nm): A, 500; B, 500; C, 500; D, 100.

A previous QFDE study (Arakawa et al. 2022) analyzed cortical ER in the filamentous fungus *Myelochroa leucotyliza*, a lichenogenic species that, in response to soluble signals from its partner alga, elaborates an apparently continuous sub‐plasmalemmal envelope (Figure 4A), much like the nuclear envelope around the nucleoplasm, from which it secretes hydrophobic compounds into the lichen extracellular matrix. The ER and plasma membrane surfaces are interconnected by prominent tethers (Arakawa et al. 2022; Saheki and De Camilli 2017; Zaman et al. 2020) which, in yeast, include three protein families: VAMP‐associated proteins (VAPs), Anoctamin/TMEM16/Ist2p homologs, and extended synaptotagmins (E‐Syts) (Gallo et al. 2016; Manford et al. 2012; Zaman et al. 2020).

Most of the microalgae evaluated in this study, including the red and complex lineages, do not display a cortical ER. Our examples derive from green microalgae: two Chlorophytes (*Polytomella parva* (Figure 4B) and *Borodinellopsis texensis* (Figure S5C)) and several Trebouxiophytes (*Auxenochlorella protothecoides* (Figure 4C), *Asterochloris glomerata*, *Trebouxia jamesii*, *Trebouxia decolorans* (Figure S5B), Trebouxia sp. (Figure S5A), and *Botryococcus braunii* (Figure S5D)). All but *B. braunii* also form plasma membrane differentiations called eisosomes (Lee et al. 2015), and proteins linking eisosomes to cortical ER are reported in yeast (Ng et al. 2020), but most eisosome‐bearing microalgae in our studies are not observed to form cortical ER (Lee et al. 2015).

Unlike the continuous envelope formed by the fungus (Figure 4A), the cortical cisternae of green microalgae adopt a reticulated architecture, sending out flat narrow rivulets to the periphery (Figure 4B,D; Figure S5A–C), the result being that the plasma membrane is a patchwork of “ER‐associated” and “ER‐non‐associated” domains. Tethers are present between the reticulate branches and the plasma membrane (Figure 4D and Figure S5C) but they are not as prominent as in *M. leucotyliza* (Arakawa et al. 2022).

Our samples include two cases where the formation of cortical ER is induced: it develops when *P. parva* undergoes encystment (Figure 4B) and when *A. protothecoides* is exposed to decane (Figure 4C). Hence, cortical ER may form in additional microalgae under untested conditions.

A second pattern is found in *Botryococcus braunii* (Hirose et al. 2013) which constructs a cortical ER carrying fenestrae (holes) (Weiss et al. 2012) (Figure S5D), a configuration also described in yeast and animals (Shemesh et al. 2014; Schroeder et al. 2018). Both ER membranes participate in generating the boundaries of a fenestrum, much like the openings that are created to contain nuclear pore complexes. In *Botryococcus*, the holes contain unidentified granular material (Figure S5D). 

In yeast and animal cells, cortical ER has been proposed to mediate the transfer of ER‐synthesized lipids to the plasma membrane (English et al. 2009; Wu, Carvalho, and Voeltz 2018). Since most of the microalgae analyzed in the present study lack a dedicated cortical ER system, they presumably accomplish this transfer by some other means.

We earlier described nuclear bridges that mark the junction between the nuclear envelope and cytoplasmic ER (Figure 2, paired dots), noting that particular bridges appear to be Golgi‐targeted and suggesting that each bridge may mark a particular pan‐cellular destination. This suggestion is supported by the bridge shown in Figure 4B (paired dots), which is fully confluent with the cortical ER included in the fracture and is thus presumably continuous with the full cortical system.

## 10 Chloroplast Endoplasmic Reticulum

The “primary” chloroplast of green and red microalgae derives from an endosymbiotic cyanobacteria that was ingested and “domesticated” by a eukaryotic host (Archibald 2015; Sibbald and Archibald 2020). Modern primary chloroplasts are surrounded by two membranes—the inward‐facing M1 that carries the TIC subunit of the chloroplast translocon, and the outward‐facing M2 that carries the TOC subunit—and they collectively form the chloroplast envelope. Modern cyanobacteria are also enclosed by two membranes, the inner containing homologs of TIC and the outer containing homologs of TOC. Hence, M1 and M2 presumably derive from their cyanobacterial antecedents, albeit many eukaryote‐specific components have been added.

A “secondary” chloroplast derives from a red or green alga that was ingested and domesticated by a second host cell. In several cases, a secondary alga has subsequently been domesticated by a third host cell to create “tertiary” lineages. Since the full ancestry of these radiations has not yet been resolved (Burki et al. 2020; Sibbald and Archibald 2020; Strassert et al. 2021), we refer to them all as “complex.” Our examples all derive from lineages with a red algal ancestor.

Each complex chloroplast is surrounded by its original M1 and M2 membranes and then by a second envelope comprised of membranes M3 (M2‐facing) and M4 (cytoplasm‐facing). The M3/M4 envelope was originally called the chloroplast ER (cpER) because of its clear ultrastructural continuity with the nuclear envelope (Gibbs 1962), a continuity documented in many additional studies (Bouck 1965; Brown and Bouck 1973; Gibbs 1979; Slankis and Gibbs 1972).

Since then, alternative schemes have been proposed (Ball et al. 2015; Burki 2017; Flori et al. 2016; Kawachi et al. 2021; Penot et al. 2022), many influenced by Cavalier‐Smith's (2000) suggestion that M3 derives from the plasma membrane of the original algal endosymbiont and M4 from the host phagosomal membrane. Gould, Maier, and Martin (2015), however, note that M3 contains SELMA, a derivative of the ERAD transporter that removes misfolded proteins from the ER lumen (Lau et al. 2015; Stork et al. 2013), leading them to support Gibb's interpretation. In their model, the standard S61 ER transporter brings nuclear‐encoded proteins across M4 into the cpER lumen; SELMA mediates the transport of these proteins across M3 to the TOC/TIC translocon spanning M2/M1, which then delivers them to the chloroplast interior. Our images are fully concordant with this model.

In the sections below we first briefly describe the QFDE faces of the M1 and M2 membranes in primary chloroplasts, which are indistinguishable in primary and complex microalgae. We then examine M3 and M4, documenting that they are indeed ER membranes. Finally, we describe the invariant relationship that forms between cpER and nuclear envelope membranes to create a junction between the iNM and the M3 membrane.

### 10.1 Primary Chloroplast Envelope

Figure S6 shows images of the M1 and M2 membranes that surround the chloroplast thylakoids to form the primary chloroplast envelope in *C. reinhardtii* . In land plants, the two membranes have distinctive proteomes (Gutierrez‐Carbonell et al. 2014; Bouchnak et al. 2019). M1 is enriched in MGDGs and phosphatidyl glycerol, as are the thylakoids, whereas M2 is enriched in DGDGs and phosphatidylcholine. M1 carries a dense IMP population on its P face (Figure S6A,B), akin to its cyanobacterial forebear (Samuel et al. 2001; Liberton et al. 2006), while M2 carries few IMPs on either face (Figure S6E).

We noted earlier that the E faces of ER‐derived membranes are occasionally “quilted” (Figures 1 and S1). The E face of M2 proves to be particularly prone to adopt this configuration in microalgae (Figure S6B,D); it is also seen in a multicellular red alga (Bisalputra and Bailey 1973). A second diagnostic feature of the M2 E face is the occasional presence of angular discontinuities that we call pits (Figure S6A,C,D). These features, observed in both primary and complex lineages, may reflect the large galactolipid endowment of M2 (Inoue 2007), which would generate a more water‐rich bilayer that is more prone to disruption by etching. The pits are also useful in distinguishing M2 from the M3/M4 membranes of the cpER.

### 10.2 The Chloroplast ER (cpER)

**FIGURE 5:**
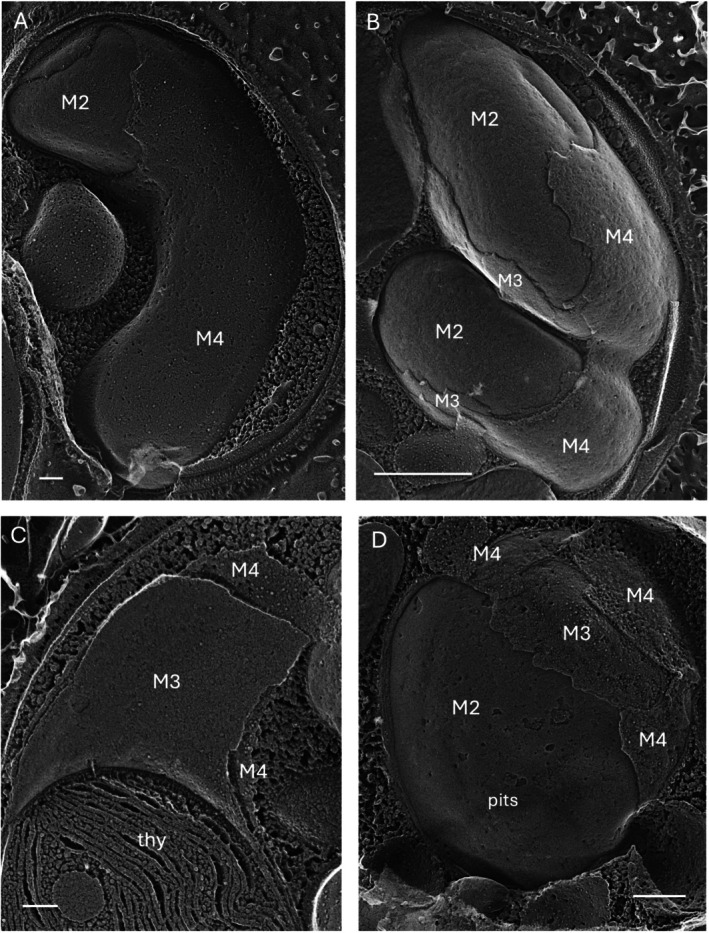
Chloroplast ER in *Nannochloropsis*. (A) *N. gaditana*. (B) *N. salina*. (C) *N. gaditana*. (D) *N. oceanica*. M2, outer membrane of chloroplast envelope; M3 and M4, inner and outer membranes of cpER; thy, thylakoids. Bars (nm): A, 100; B, 500; C, 100; D, 250.

Figure 5 shows images of the M3/M4 membranes that encase the M1/M2 envelopes in the complex microalga *Nannochloropsis*, whose bilayers are particularly prone to generate long fracture faces. The M4 membrane carries ribosomes while the M3 membrane does not (Bouck 1965; Gibbs 1981). M3 is in close contact with the M2 membrane, reminiscent of the cortical ER in association with the plasma membrane, although tethers are not evident. In some cross‐fractured images, all four membranes are directly contiguous with their lumens obscured (Figure S7A). The M3/M4 fracture faces display no features that distinguish them from other regions of the ER.

The dinoflagellates provide an exception to the rule that complex plastids derived from red algae are enveloped by a cpER cisternum. Some images of chloroplasts in *Symbiodinium* (Figure S7B) and *Gonyaulax* (Herman and Sweeney 1975) show a canonical M1/M2 envelope with no surrounding cpER, while others show an ER only loosely associated with the envelope (Figure S7C) or a single membrane surrounding the envelope (Dodge 1968). This feature appears to be unique to the dinophyte radiation of the Alveolata, however, since the photosynthetic apicomplexan‐related radiation of the Alveolata, exemplified by *Chromera*, preserves a classic M3/M4 arrangement (Figure S7D; Obornik and Lukeš 2013), as does the remnant apicoplast in parasitic apicomplexan radiations (Figure S7E; Lemgruber et al. 2013; Parsons et al. 2007). Given the wide range of evolutionary innovation that the dinoflagellates have explored http://www.tolweb.org/Dinoflagellates/2445, it may be that they have also experimented with modes of chloroplast management.

As first shown in thin‐section images (Gibbs 1979; Hibberd and Leedale 1972; Manton 1966; Slankis and Gibbs 1972), M3 loses its close association with M2 in localized domains, creating a narrow gap populated by membranous profiles that are frequently vesicular. The lumen between M3 and M4 also tends to dilate in these regions, which we designate as Gibbs domains (Gibbs 1979). Figure S8 shows their QFDE morphology. Gibbs (1979) suggests that they represent regions engaged in cargo transport from the cpER to the chloroplast, positing M3 vesiculation and vesicle/M2 fusion as the delivery pathway, a pathway perhaps independent of a SELMA‐mediated pathway (Gould, Maier, and Martin 2015). Gibbs domains often localize at the chloroplast midline (Figure S8A) suggesting that they may play a role in determining chloroplast size or division parameters.

### 10.3 Junctional ER

**FIGURE 6:**
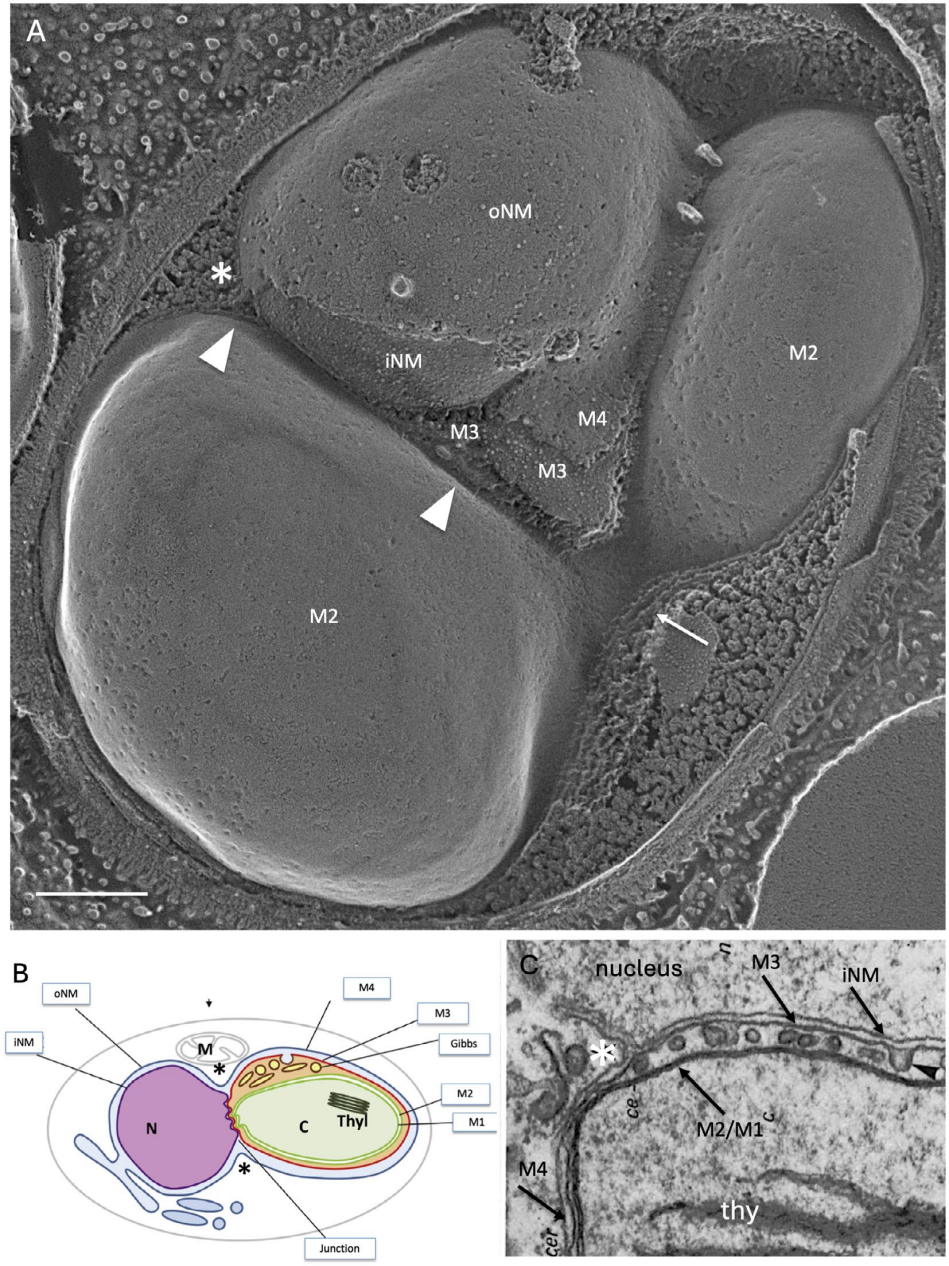
Chloroplast‐nuclear junctions. (A) A nucleus forms junctions with two lobes of a dividing chloroplast in *N. gaditana*. The lower junction, its visible portion bracketed by arrowheads, entails formation of junctional ER between the INM and M3. Asterisk marks the extension between the oNM and M4. (B) Diagram of a chloroplast‐nuclear junction (after Flori et al. 2016). (C) Thin‐section of *O. danica* (Gibbs 1981) showing a Gibbs domain subtending a junction. arrow, bridge connecting dividing chloroplast; asterisk, oNM transition to M4; iNM and oNM, inner and outer membranes of nuclear envelope; M1and M2, inner and outer membranes of chloroplast envelope; M3 and M4, inner and outer membranes of cpER; thy, thylakoid. Bar (nm): A, 250.

**FIGURE 7:**
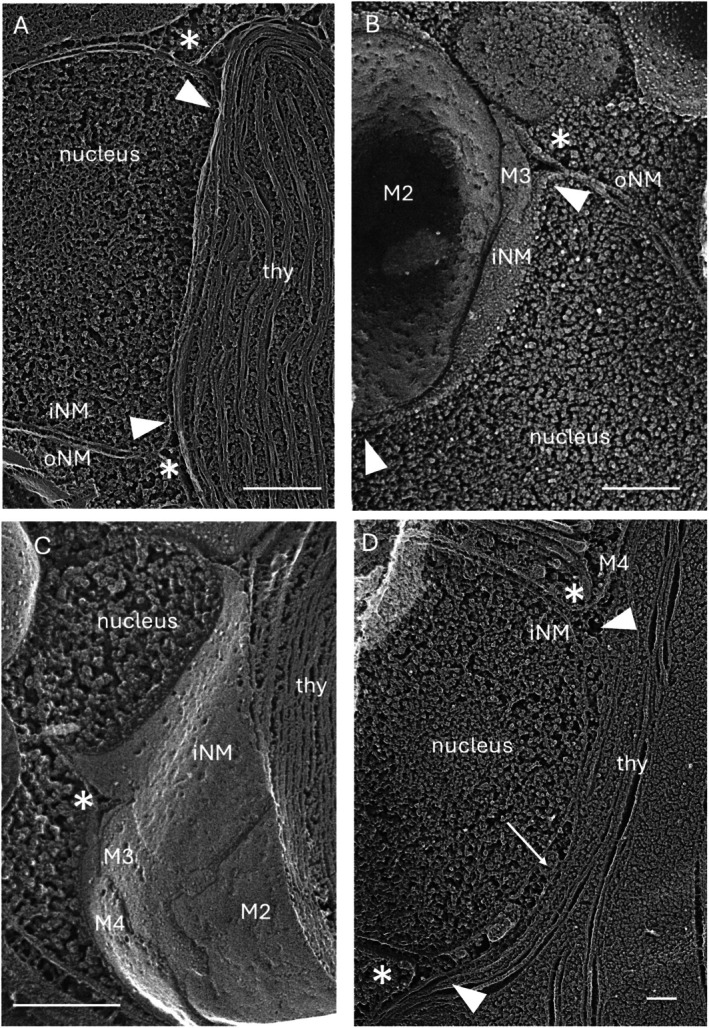
Chloroplast‐nuclear junctions. (A) *Ochromonas danica* (B) *Nannochloropsis salina* (C) *N. salina* (D) *Phaeodactylum tricornutum*. See legend to Figure 6. Arrow, material between iNM and M3. Bars (nm): A, 500; B, 250; C, 250; D, 100.

As noted earlier, the convergence of most forms of cytoplasmic ER with the nuclear envelope is marked by narrow protrusions of the oNM that we call nuclear bridges (Figure 2). A distinctive and unique topology is adopted at the convergence of the cpER and the nuclear envelope (Figures 6 and 7). The nucleus and chloroplast become juxtaposed over a distance of 1–2 μm, a configuration designated the chloroplast‐nuclear junction (Slankis and Gibbs 1972). At the periphery of these juxtapositions, the ribosome‐studded oNM disassociates from the iNM and extends out to become continuous with the ribosome‐studded M4 of the cpER, extensions marked with asterisks in Figures 6 and 7. The abandoned iNM instead establishes direct contact with the exposed M3 membrane along the length of their juxtaposition, as bracketed by arrowheads in Figures 6 and 7. M3 then joins M4 at the periphery to collectively encircle the plastid, while the iNM re‐joins the oNM to collectively encircle the nucleus. At the time of plastid division, a second such junction is formed (Figure 6A).

The heart of the chloroplast‐nuclear junction, then, is a hybrid segment of ER, with the iNM side facing the nucleoplasm and the M3 side facing the chloroplast envelope, neither carrying ribosomes. We call this segment the junctional ER. Formally, this configuration is the same as that adopted by the nuclear Golgi (Figure 3), where one side faces the nucleoplasm and the other faces the cis elements of the Golgi.

Chloroplast‐nuclear junctions are evident in thin‐section images of numerous complex lineages, although they are often not noted (Bouck 1965; Dodge 2012; Falk and Kleinig 1968; Flori et al. 2016; Fresnel and Probert 2005; Gibbs 1979; Kawachi et al. 2021; Manton 1966). Flori et al. (2016) lift up the iNM/M3 pairing in a diagram, re‐drawn here as Figure 6B, where they offer a different interpretation of the participating membranes.

Membranous profiles may occupy a gap between M3 and its underlying M2 in the junction region (Figure 6C), an arrangement identical to that described earlier for the Gibbs domains in nonnuclear regions (Figure S8). Material may also occupy a gap between the iNM and M3 (Figure 7D). Hence, the junctions are positioned to associate with cargo that may be moving between the nucleoplasm and the chloroplast interior.

### 10.4 Cryptophyte Organization

**FIGURE 8:**
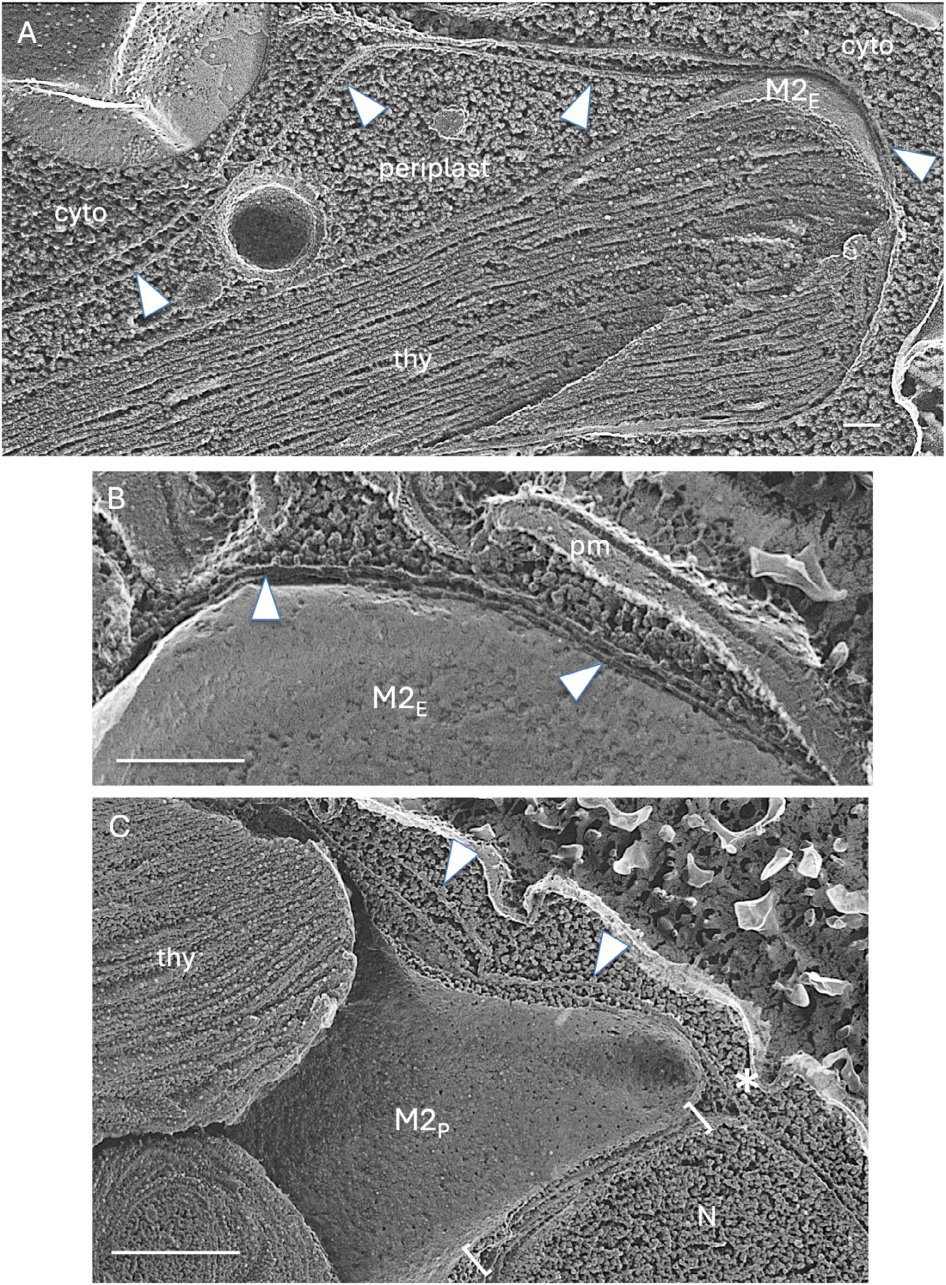
PeriER in *Chroomonas mesostigmatica*. (A) PeriER (arrowheads) creates a periplasm and associates with the chloroplast envelope at right. (B) PeriER closely associated with the chloroplast. (C) Chloroplast‐nuclear junction. Arrowheads, PeriER; asterisk, extension between the oNM and the cytoplasm‐facing membrane of the periER; brackets, chloroplast‐nuclear junction; cyto, host cytoplasm; M2_E_, E face of the outer chloroplast envelope membrane; M2_P_, P face of the outer chloroplast envelope membrane; N, nucleus; pm, plasma membrane. Bars (nm): A, 100; B, 250; C, 500.

The cryptophyte algae (Hoef‐Emden and Archibald 2016) are widely regarded as the modern direct descendants of the founding lineage that first engulfed and domesticated a primary red alga to form a complex alga. Cryptophytes display two cytoplasmic domains (Figure 8A). The host‐derived domain harbors the nucleus of the original host and its attendant organelles (mitochondria, flagella, etc.); the algal symbiont‐derived domain, called the periplast, harbors the chloroplast, its cytoplasmic starch products, and a nucleomorph, a remnant version of the domesticated alga's nucleus (Zauner et al. 2019), which encodes the SELMA protein proposed to mediate transport from the chloroplast ER to the plastid (Gould, Maier, and Martin 2015). A membrane cisternum defines these two domains, one side facing the host‐derived cytoplasm and bearing ribosomes (Hill and Wetherbee 1986; Laza‐Martínez et al. 2012; Figure S9A) and the other facing the periplast and ribosome‐free (Figure 8A arrowheads). The continuity of this cisternum with the nuclear envelope, documented in Figure S9A,B, establishes it as an ER cisternum that we designate as periER. The periER serves as the host/periplast boundary in some regions (Figure 8A) while in other regions it makes close contact with the chloroplast envelope M2 (Figure 8A,B), serving as an M3/M4 equivalent.

Topologically, the periplast occupies the same domain as the Gibbs domains (Figure S8)—sandwiched between the periER and the M2 of the chloroplast envelope—leading to the proposal that the Gibbs domains represent vestigial periplasts (Falk and Kleinig 1968; Grosche et al. 2014).

Importantly, chloroplast‐nuclear junctions are also found in the cryptophytes. Figure 8C shows a junction in *Chroomonas*, and thin‐sectioned images from the published literature, presented in Figure S9, document their ubiquity throughout the cryptophyte radiation; indeed, they are even included, although not pointed out, in diagrams of cryptophyte morphology (Figure S9B). Hence, cryptophytes produce a periER that envelops the plastid like the M3/M4 ER and participates in canonical chloroplast‐nuclear junctions. This finding suggests that the domestication of the first red algal chloroplast may have involved creating chloroplast‐nuclear junctions, a differentiation that has persisted for hundreds of millions of years of complex algal radiation.

## 11 Perspectives

The guiding dynamic in preparing this review has been to allow the reader to experience the topography and fine structure of the ER and associated organelles in three dimensions. The images have, in turn, lifted up questions as to how this very long membrane, folded back on itself to create a continuous tiny lumen, is able to segregate its various functions, correctly localize in the cell, and acquire novel functions in particular lineages. We conclude by considering three of these questions.

### 11.1 How Does the ER Establish Localized Domains That Perform a Different Function Than the Surrounding Bilayer?

The canonical example of this trait is the Golgi, where an ER domain, 500 nm–1 μm in *Chlamydomonas*, becomes differentiated from the adjacent rough ER and associates with coats to deliver membrane and cargo to the Golgi stacks (Figure S3). A thin‐section illustration of this arrangement is shown in Figure S3B, where a ribosome‐free length of the ER faces the Golgi. The oNM can also service Golgi stacks in microalgae to create nuclear Golgi (Figure 3), meaning that it can also become re‐programmed.

How does this work? Do pre‐synthesized Golgi stacks “recruit” ER membrane to shed its ribosomes and differentiate into a partner membrane, or does the ER assemble a domain capable of creating or partnering with Golgi? The differentiation is presumably not dictated by soluble materials in the lumen since the membrane on the other side of the lumen continues to bear ribosomes. Spatial patterning in the plasma membrane is known to be influenced by associations with membrane‐binding proteins such as the epiplastins (Goodenough et al. 2018), but no such systems are known to associate with microalgal ER membranes. In short, nothing is known.

Golgi and lipid body partnerships engage restricted regions of an ER membrane. Partnerships can also involve one whole side of a cisternum: the association of the iNM with chromatin; the association of the cortical ER with the plasma membrane; and the association of the chloroplast ER (cpER) M3 membrane with the M2 membrane in the chloroplast envelope. The same question pertains here: do the partners have “ER‐seeking” displays or does the ER have “partner‐seeking” displays, or are both operant?

In the above cases, the side opposite a differentiated membrane continues to bear ribosomes. In two additional cases, neither side binds ribosomes: the ER of the nuclear Golgi faces Golgi stacks on one side and ribosome‐free iNM on the other, and the junctional ER faces cpER M3 on one side and iNM on the other.

This raises a general unanswered query: do apposing sides of a cisternum “know” what the other side is doing, or do they operate independently? For example, are the ribosomes on the opposite side of a Golgi‐servicing membrane enriched for ribosomes translating Golgi‐related proteins? Are nucleus‐targeted glycoproteins (Comer and Hart 2000) favored in the case of the nuclear Golgi? Are the ribosomes on cortical ER enriched for mRNAs encoding nonglycosylated plasma membrane and secreted proteins? There are reports of localized translation in other systems (Lerit 2022; Vasek et al. 2023), but none as yet implicate the ER.

### 11.2 How Does the ER “Know Where to Go” in a Microalgal Cell?

In fungi, plants, and animals, microtubule‐ and/or actin‐based cytoskeletons subtend the pathways for ER migration (English et al. 2009; Friedman and Voeltz 2011; Kontou et al. 2022; Obara et al. 2024; Westrate et al. 2015; Sparks et al. 2011; Wu, Carvalho, and Voeltz 2018; Zang et al. 2021). However, microalgal cytoplasms contain no microtubule/actin cytoskeletons. Hence, directionality must somehow be programmed into the ER system itself, where possibly this information proceeds to recruit cytoskeletal associates in nonalgal organisms.

We suggest that the nuclear bridges between the nuclear envelope and cytoplasmic ER may be endowed with such information. In this scenario, each bridge is programmed to endow its coextensive cisternae with core identifiers as to location and function, identifiers that may be further refined as the membrane traverses the cytoplasm. If this were the case, then the nuclear envelope could be considered a hub that sends out differentiated cisternal/tubular spokes and thereby contributes to the organization of the cytoplasm.

### 11.3 How Does the ER Acquire New Functions?

We describe here a core set of ER functions—protein synthesis, nuclear maintenance, Golgi interactions, lipid body synthesis, plasma membrane associations, and nuclear bridge formation—that are found throughout the eukaryotes and hence were presumably operant in the LECA. In multicellular lineages, the ER is often recruited to participate as well in specialized functions, like the sarcoplasmic reticulum in muscle cells.

Complex microalgae harbor such a specialization in the form of the M3/M4 cpER, which has two features: it envelops the primary chloroplast envelope (M1/M2), and its M3 membrane forms a unique hybrid with the iNM to create a junctional ER that anchors the two organelles together in a chloroplast/nuclear junction. This arrangement is very different from the bridges, formed solely by the oNM, that connect the nuclear envelope with other versions of the ER.

Of particular interest is that chloroplast/nuclear junctions are found in cryptophytes, considered to be “living fossils” of the original organisms that domesticated a red alga. That they have persisted through all the subsequent complex “red” radiations (except dinoflagellates) indicates their salience. We therefore propose that they played, and continue to play, a foundational role in the domestication process. For example, since each cell forms a single junction and forms a second at the time of plastid division (Figure 6A), it may play a role in maintaining the correct number and size of the organelles. There is also evidence of materials sequestered within the junction (Figures 6C and 7D) that may represent cargo or informational molecules exchanged between the two organelles. Future models of complex algal dynamics will hopefully be enriched by this discovery.

## Supporting information


**Figure S1.** Nuclear envelopes and their pores in complex algae and fungi. (A) *Nannochloropsis gaditana*. (B) *Eustigmatos vischeri*. (C) *Pelagomonas calceolata.* (D) Diatom endosymbiont of dinotom *Glenodinium foliaceum* . (E) Fungus *Cladonia grayi* . (F) Fungus *Saccharomyces cerevisii*. I_E_, inner membrane E face; I_P_, inner membrane P face; O_E_, outer membrane E face. Bars (nm): A, 250; B, 500; C, 250; D, 250; E, 250; F, 100.

**Figure S2.** Cisternal faces of algal cytoplasmic ER. (A) *Nannochloropsis gaditana*. (B) *Pelagomonas calceolata*. (C) *N. oceanica*. (D) *N. oceanica*. (E) *Auxenochlorella protothecoides.* (F) *Thalassiosira* pseudonana. asterisk, aligned row of ribosomes; E, E face; P, P face; v, vacuole membrane. Bars (nm): A, 500; B,100; C, 250; D, 250; E, 250; F, 500.

**Figure S3.** ER/Golgi relationships in *Chlamydomonas reinhardtii* (A) Freeze‐substituted thin‐sectioned preparation; four Golgi, an unidentified black material marking cisternae and vesicles. (B) Thin section of an encysting *C. reinhardtii* 6 h zygote (Minami and Goodenough 1978). (C) Three Golgi. (D) Coated vesicles off Golgi (yellow). (E) Single Golgi. asterisks, ER lumen; dG & pG, Golgi stacks, bloated with cyst wall fibrils, proximal and distal to the ER; E, E face of ER; m, mitochondrion; N, nucleus; rER, rough ER. Bars (nm): A, 500; C, 250; D, 250; E, 500.

**Figure S4.** ER/lipid body relationships. (A) *Chlamydomonas reinhardtii*. (B) *Galdieria sulfuraria*. (C) *Ochromonas danica*. (D) *Cyanidioschyzon merolae*. (E) *Botryococcus braunii* showing nuclear envelope associated with two lipid bodies, cytoplasmic ER‐associated with a third. (F) *Nannochloropsis salina* showing IMP‐free ER extending over lipid body (arrow). (G) *C. reinhardtii* showing cisternal and tubular ER elements. asterisks, cisternal ER; L, lipid body; N, nucleus; t, tubular ER. Bars (nm): A, 250; B., 250; C, 500; D, 250; E, 500; F, 100; G, 250.

**Figure S5.** Reticulated and fenestrated cortical ER. (A) *Trebouxia* sp. in *Myelochroa leucotyliza* lichen (see also Arakawa et al. 2022). (B) *Trebouxia decolorans* in *Candelaria* lichen (see also Goodenough and Roth 2021, Figure 5). (C) *Borodinellopsis texensis*. (D) *Botryococcus braunii*. arrowhead, tethers between pm and ER; E, E face of ER membrane; ei, eisosome; P, P face of ER membrane; pm, plasma membrane; s, secretion pore. Bars (nm): A, 250; B, 500; C, 250; D, 100.

**Figure S6.** Chloroplast envelope of *Chlamydomonas reinhardtii* . M1_P_, P face of inner envelope membrane (M1); M2_E_, E face of outer envelope membrane (M2); M2_P_, P face of outer envelope membrane; pits and quilts, textures of M2_E_; s, starch; thy, thylakoids. Bars (nm): A, 500; B, 250; C, 250; D, 500; E, 500.

**Figure S7.** Chloroplast ER. (A) M1‐M4 in direct contact (bracket) in *Nannochloropsis salina*. (B) *Symbiodinium* sp. (C) *Symbiodinium* sp. (D) *Chromera velia*. (E) Apicoplast in *Neospora caninum* sp. arrowheads, cpER; M2_E_, E face of outer membrane (M2) of envelope; M2_P_, P face of outer membrane (M2) of envelope; pits and quilt, textures of M2; thy, thylakoids; w, wall. Bars (nm): A, 100; B, 500; C, 500; D, 500; E, 100.

**Figure S8.** Gibbs domains (arrowheads). (A) *Nannochloropsis gaditana*. (B) *N. salina*. (C) *Ochromonas danica*. (D) *Thallasiosira pseudonana*. (E) *Ochromonasdanica*. M2_E_, E face of outer membrane (M2) of envelope; thy, thylakoids; asterisks, cross‐fractures of cpER. Bars (nm): A, 250; B, 250; C, 250; D, 250; E, 250.

**Figure S9.** Chloroplast‐nuclear junctions in published images of cryptophytes. (A) *Teleaulax ampnioxeia* (Laza‐Martinez et al. 2012). (B) *Cryptomonas* sp. (Santore 1985). (C) *Chroomonas mesostigmatica* (Dodge 1969). (D) Cryptophyte diagram (Hoef‐Emden and Archibald 2016). (E) *Chroomonas* sp. (Nam et al. 2021). (F) *Cryptomonas* sp. (Dodge 2012). arrowheads, periER; asterisks, extension between the oNE and periER; Cp, chloroplast; no & nu, nucleolus; P & Py, pyrenoid; S & St, starch; thy, thylakoids.

## Data Availability

The authors have nothing to report.
