# Supplementary figures and images for: Ultrastructure of the Endoplasmic Reticulum in Eukaryotic Microalgae
Source: J Eukaryot Microbiol. 2025 Jul 30;72(5):e70030. doi: 10.1111/jeu.70030 (PMC12309347; doi:10.1111/jeu.70030)

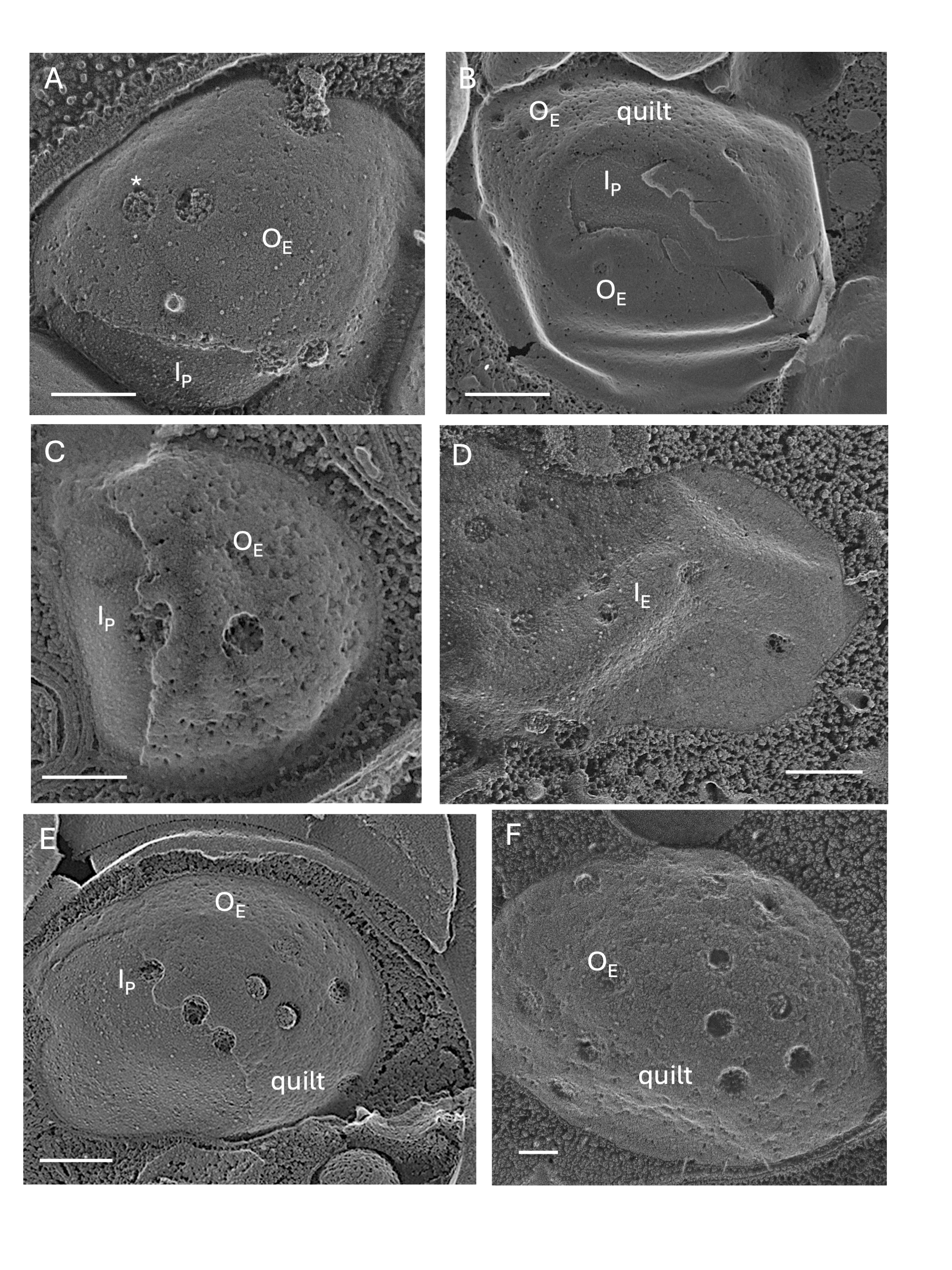

Supplement: Supplementary file 1 — Figure S1. Nuclear envelopes and their pores in complex algae and fungi. (A) Nannochloropsis gaditana. (B) Eustigmatos vischeri. (C) Pelagomonas calceolata. (D) Diatom endosymbiont of dinotom Glenodinium foliaceum . (E) Fungus Cladonia grayi . (F) Fungus Saccharomyces cerevisii. IE, inner membrane E face; IP, inner membrane P face; OE, outer membrane E face. Bars (nm): A, 250; B, 500; C, 250; D, 250; E, 250; F, 100. [file JEU-72-e70030-s002.tiff]

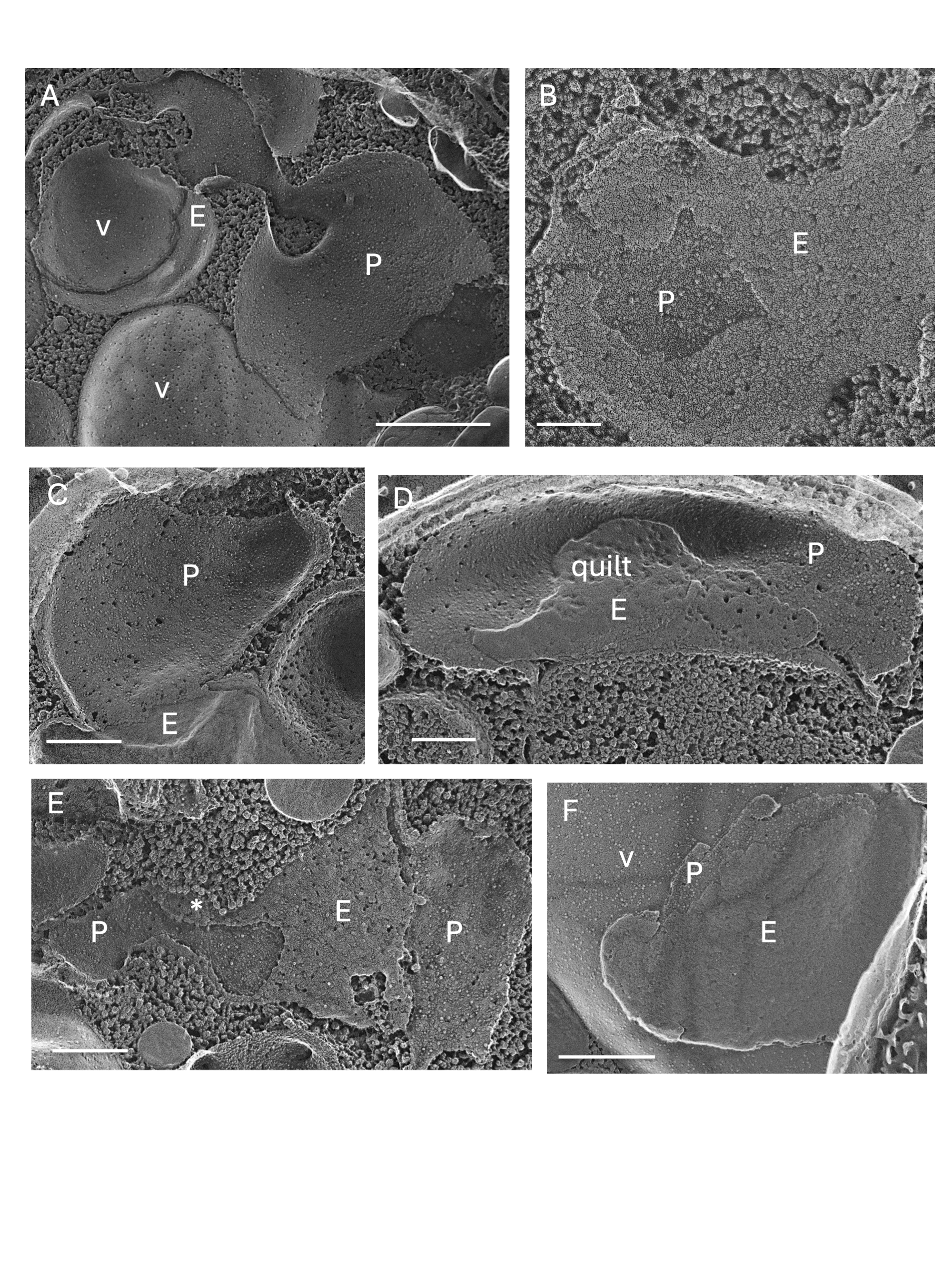

Supplement: Supplementary file 2 — Figure S2. Cisternal faces of algal cytoplasmic ER. (A) Nannochloropsis gaditana. (B) Pelagomonas calceolata. (C) N. oceanica. (D) N. oceanica. (E) Auxenochlorella protothecoides. (F) Thalassiosira pseudonana. asterisk, aligned row of ribosomes; E, E face; P, P face; v, vacuole membrane. Bars (nm): A, 500; B,100; C, 250; D, 250; E, 250; F, 500. [file JEU-72-e70030-s009.tiff]

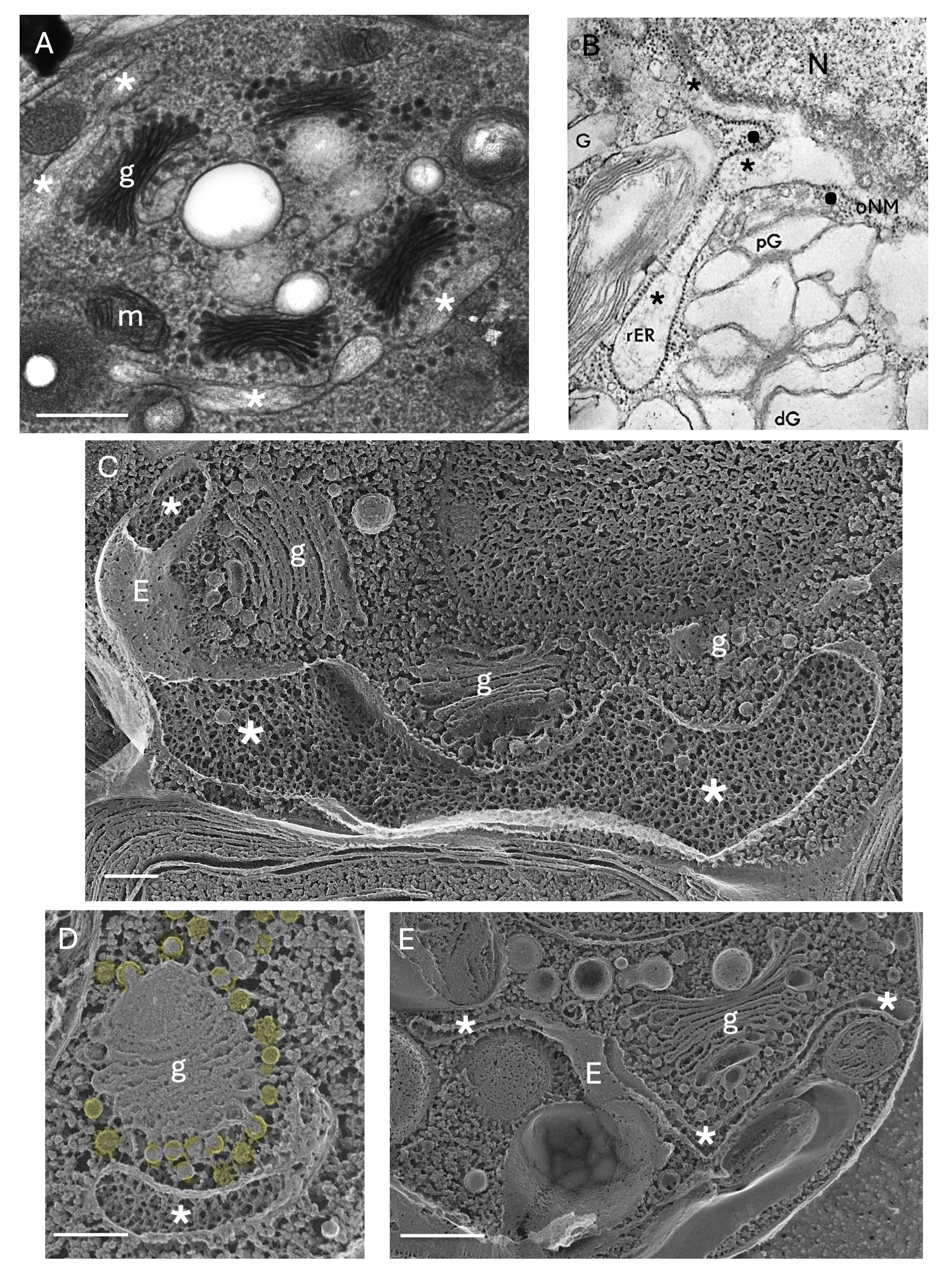

Supplement: Supplementary file 3 — Figure S3. ER/Golgi relationships in Chlamydomonas reinhardtii (A) Freeze‐substituted thin‐sectioned preparation; four Golgi, an unidentified black material marking cisternae and vesicles. (B) Thin section of an encysting C. reinhardtii 6 h zygote (Minami and Goodenough 1978). (C) Three Golgi. (D) Coated vesicles off Golgi (yellow). (E) Single Golgi. asterisks, ER lumen; dG & pG, Golgi stacks, bloated with cyst wall fibrils, proximal and distal to the ER; E, E face of ER; m, mitochondrion; N, nucleus; rER, rough ER. Bars (nm): A, 500; C, 250; D, 250; E, 500. [file JEU-72-e70030-s008.tiff]

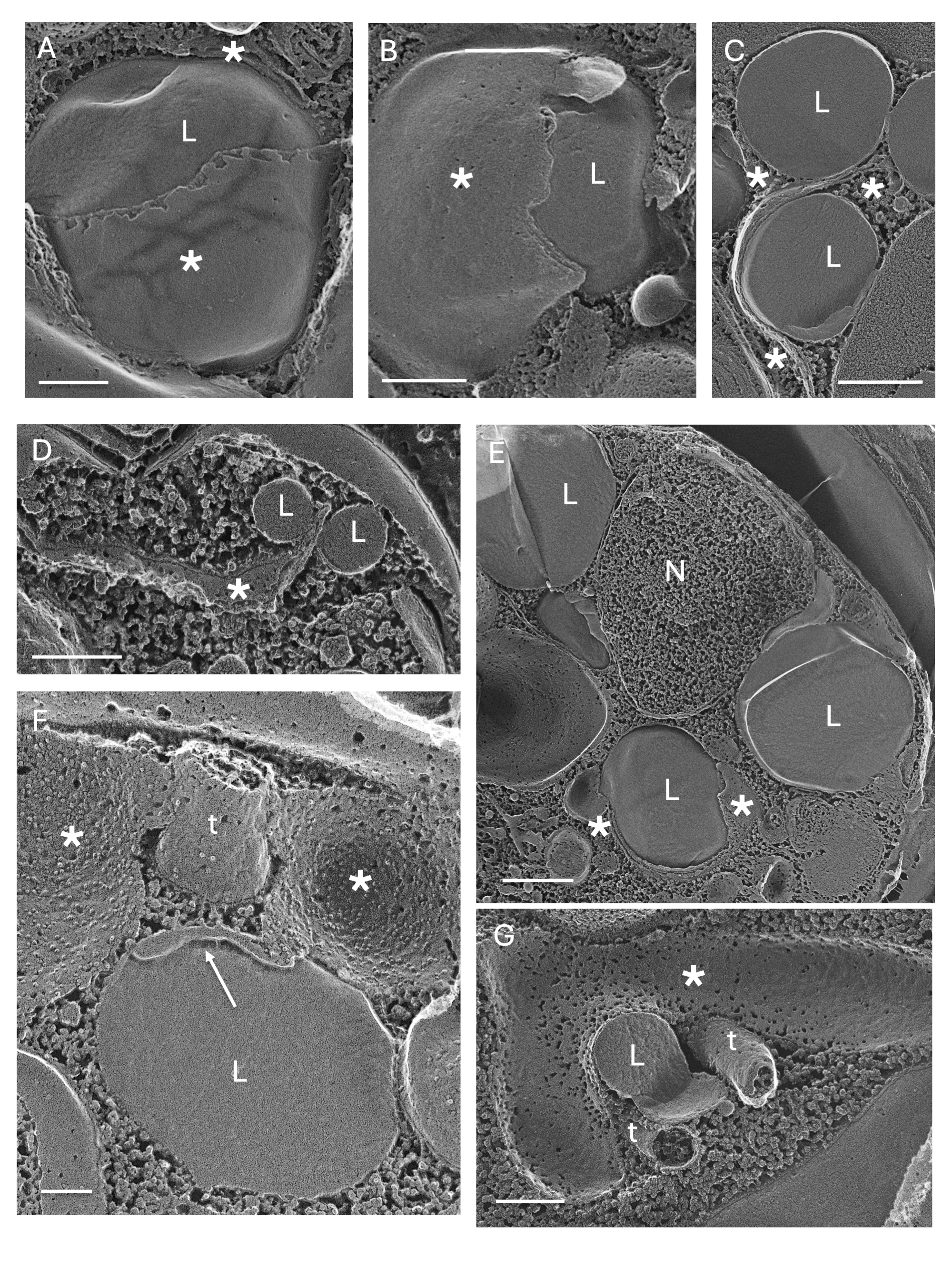

Supplement: Supplementary file 4 — Figure S4. ER/lipid body relationships. (A) Chlamydomonas reinhardtii. (B) Galdieria sulfuraria. (C) Ochromonas danica. (D) Cyanidioschyzon merolae. (E) Botryococcus braunii showing nuclear envelope associated with two lipid bodies, cytoplasmic ER‐associated with a third. (F) Nannochloropsis salina showing IMP‐free ER extending over lipid body (arrow). (G) C. reinhardtii showing cisternal and tubular ER elements. asterisks, cisternal ER; L, lipid body; N, nucleus; t, tubular ER. Bars (nm): A, 250; B., 250; C, 500; D, 250; E, 500; F, 100; G, 250. [file JEU-72-e70030-s007.tiff]

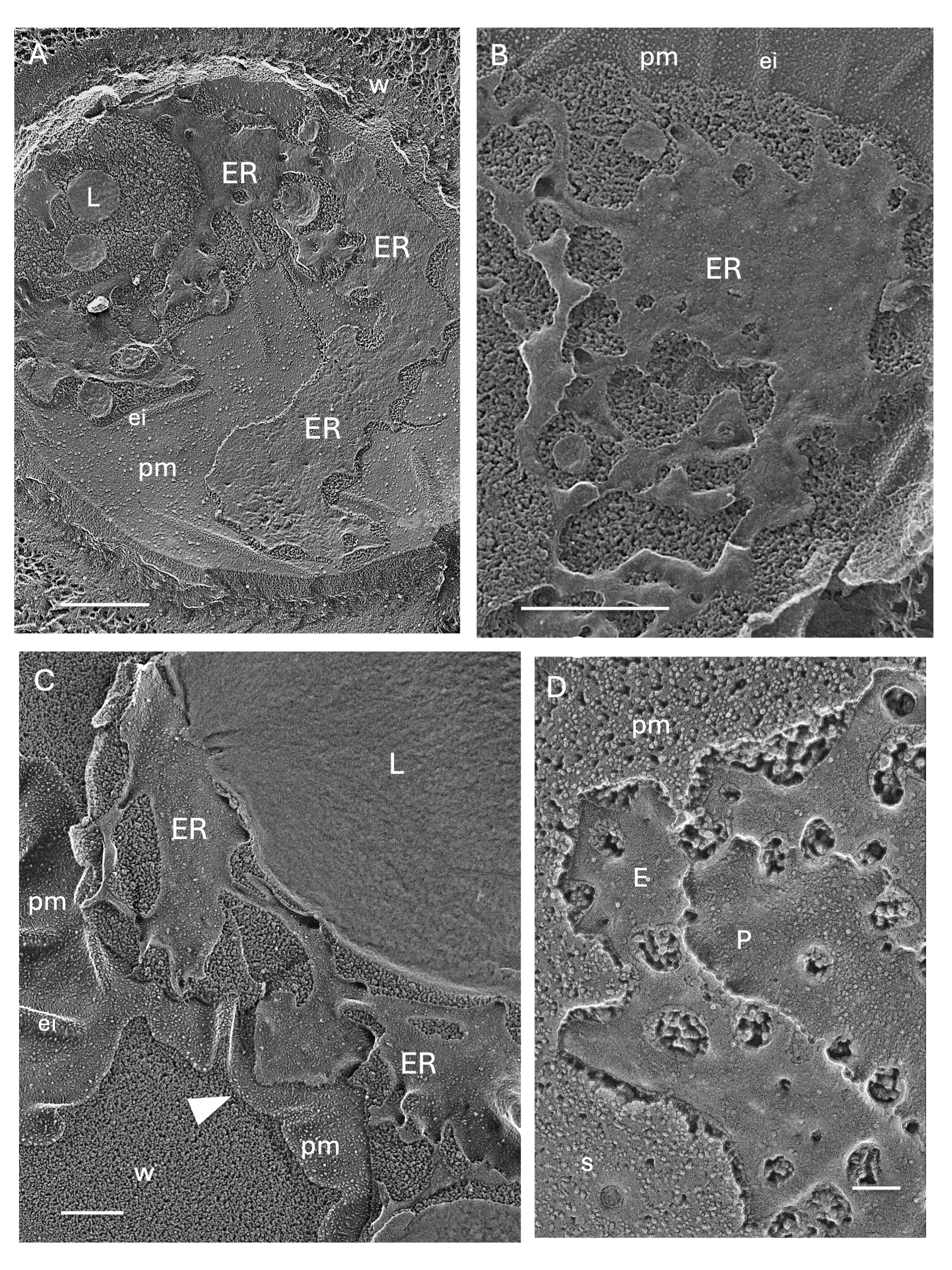

Supplement: Supplementary file 5 — Figure S5. Reticulated and fenestrated cortical ER. (A) Trebouxia sp. in Myelochroa leucotyliza lichen (see also Arakawa et al. 2022). (B) Trebouxia decolorans in Candelaria lichen (see also Goodenough and Roth 2021, Figure 5). (C) Borodinellopsis texensis. (D) Botryococcus braunii. arrowhead, tethers between pm and ER; E, E face of ER membrane; ei, eisosome; P, P face of ER membrane; pm, plasma membrane; s, secretion pore. Bars (nm): A, 250; B, 500; C, 250; D, 100. [file JEU-72-e70030-s003.tiff]

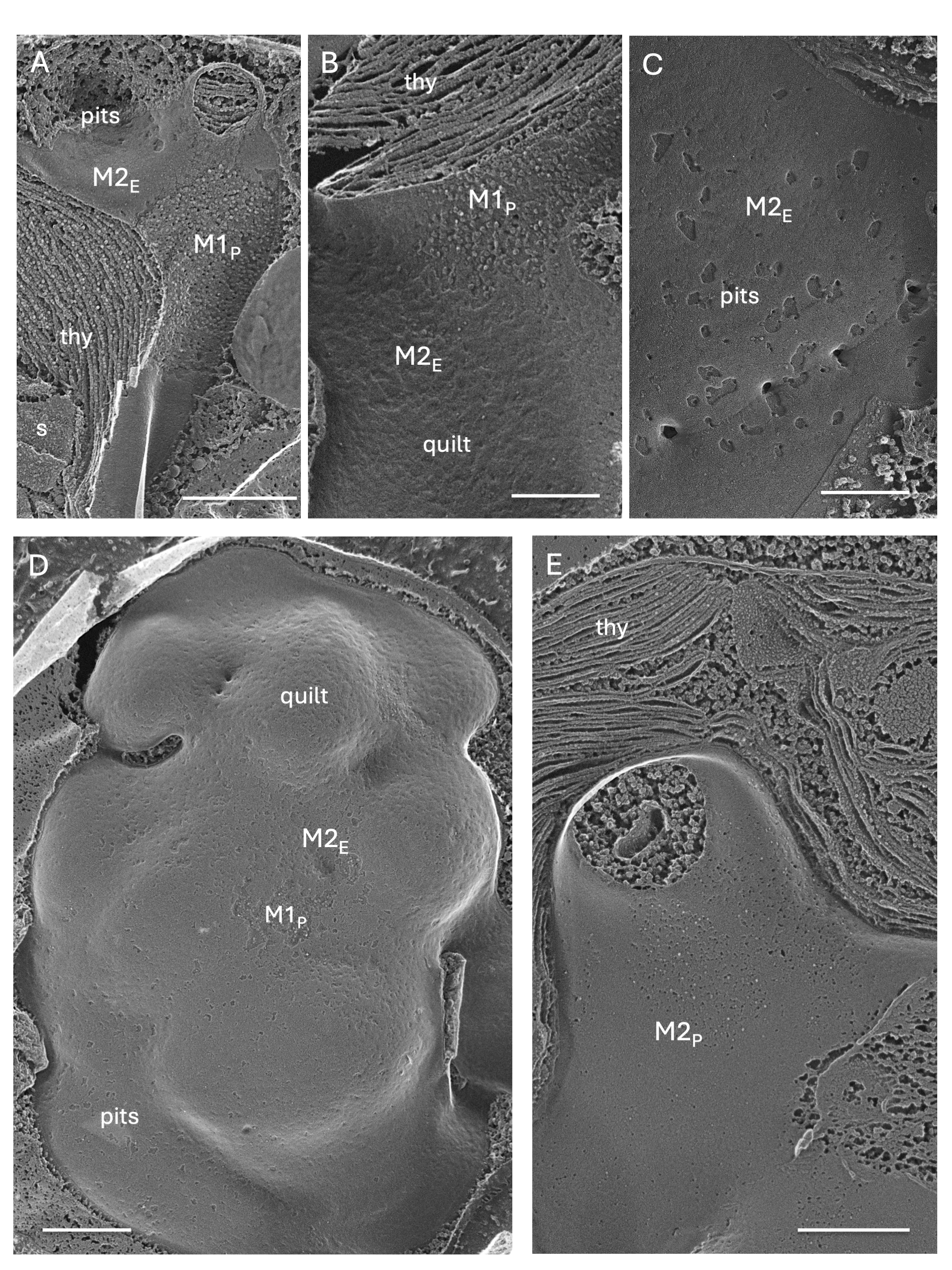

Supplement: Supplementary file 6 — Figure S6. Chloroplast envelope of Chlamydomonas reinhardtii . M1P, P face of inner envelope membrane (M1); M2E, E face of outer envelope membrane (M2); M2P, P face of outer envelope membrane; pits and quilts, textures of M2E; s, starch; thy, thylakoids. Bars (nm): A, 500; B, 250; C, 250; D, 500; E, 500. [file JEU-72-e70030-s004.tiff]

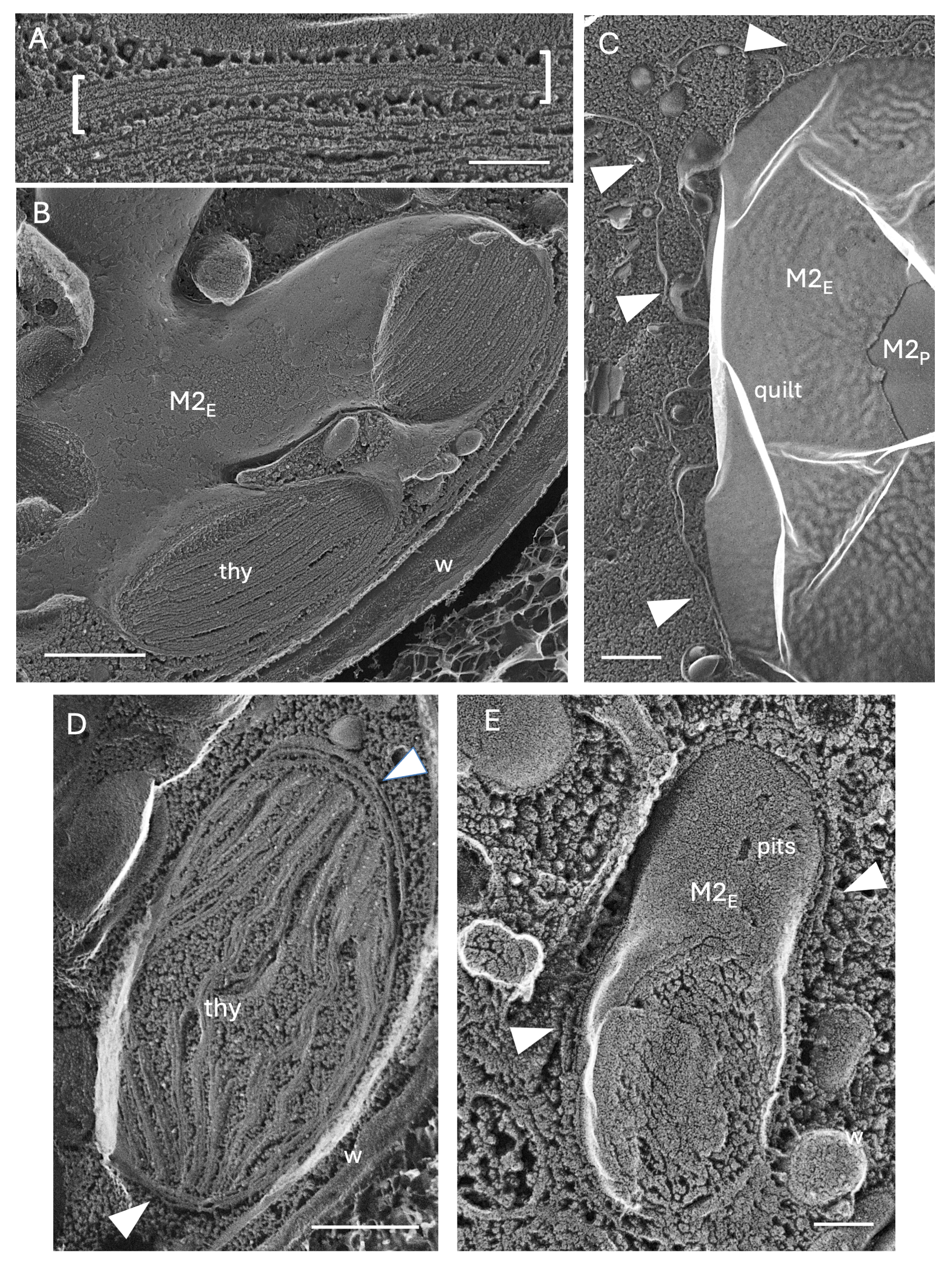

Supplement: Supplementary file 7 — Figure S7. Chloroplast ER. (A) M1‐M4 in direct contact (bracket) in Nannochloropsis salina. (B) Symbiodinium sp. (C) Symbiodinium sp. (D) Chromera velia. (E) Apicoplast in Neospora caninum sp. arrowheads, cpER; M2E, E face of outer membrane (M2) of envelope; M2P, P face of outer membrane (M2) of envelope; pits and quilt, textures of M2; thy, thylakoids; w, wall. Bars (nm): A, 100; B, 500; C, 500; D, 500; E, 100. [file JEU-72-e70030-s001.tiff]

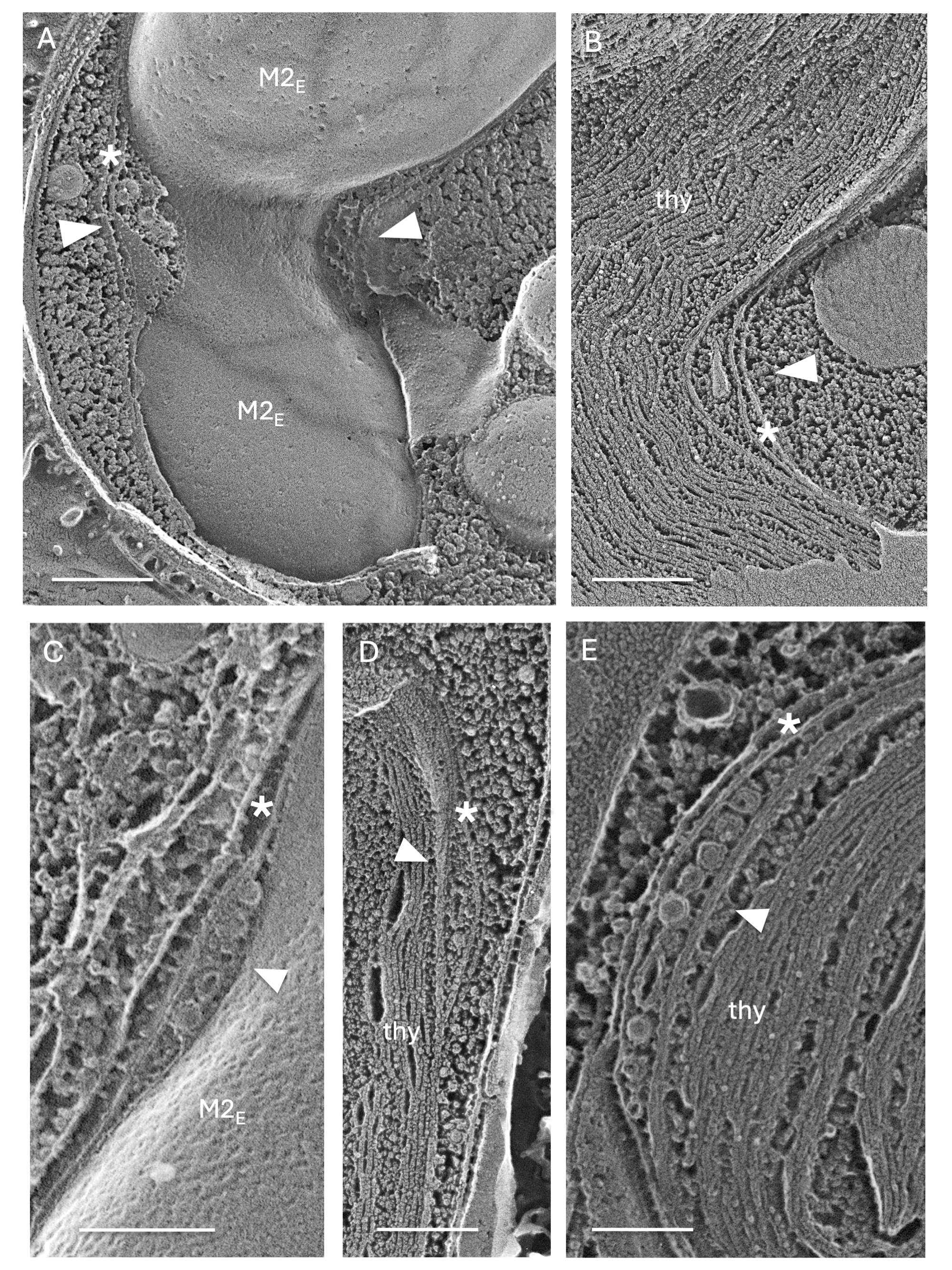

Supplement: Supplementary file 8 — Figure S8. Gibbs domains (arrowheads). (A) Nannochloropsis gaditana. (B) N. salina. (C) Ochromonas danica. (D) Thallasiosira pseudonana. (E) Ochromonasdanica. M2E, E face of outer membrane (M2) of envelope; thy, thylakoids; asterisks, cross‐fractures of cpER. Bars (nm): A, 250; B, 250; C, 250; D, 250; E, 250. [file JEU-72-e70030-s006.tiff]

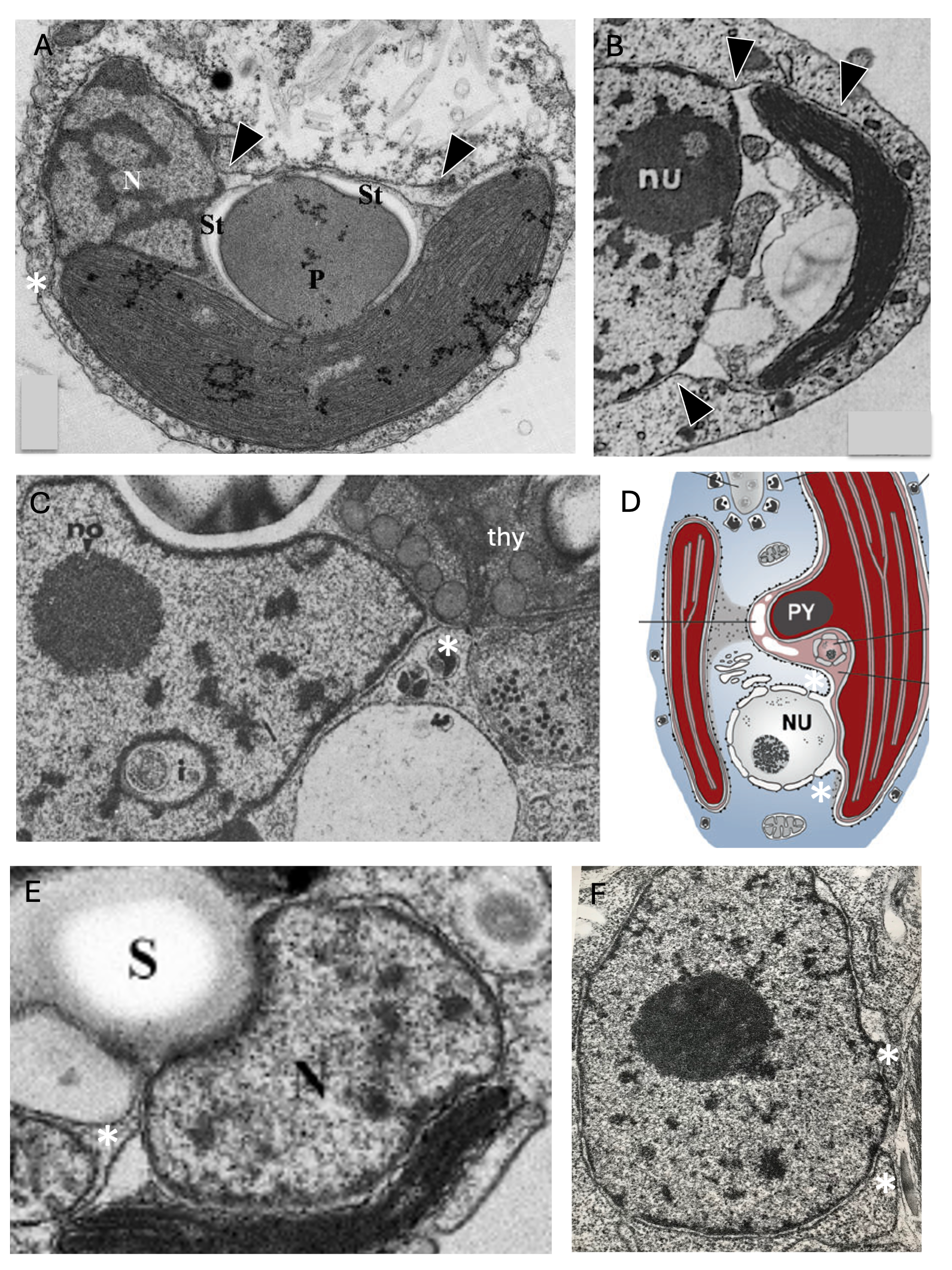

Supplement: Supplementary file 9 — Figure S9. Chloroplast‐nuclear junctions in published images of cryptophytes. (A) Teleaulax ampnioxeia (Laza‐Martinez et al. 2012). (B) Cryptomonas sp. (Santore 1985). (C) Chroomonas mesostigmatica (Dodge 1969). (D) Cryptophyte diagram (Hoef‐Emden and Archibald 2016). (E) Chroomonas sp. (Nam et al. 2021). (F) Cryptomonas sp. (Dodge 2012). arrowheads, periER; asterisks, extension between the oNE and periER; Cp, chloroplast; no & nu, nucleolus; P & Py, pyrenoid; S & St, starch; thy, thylakoids. [file JEU-72-e70030-s005.tiff]
